# Good things peak in pairs: a note on the bimodality coefficient

**DOI:** 10.3389/fpsyg.2013.00700

**Published:** 2013-10-02

**Authors:** Roland Pfister, Katharina A. Schwarz, Markus Janczyk, Rick Dale, Jonathan B. Freeman

**Affiliations:** ^1^Department of Psychology III, Institute of Psychology, Julius Maximilians University of WürzburgWürzburg, Germany; ^2^University Medical Center Hamburg-EppendorfHamburg, Germany; ^3^Cognitive and Information Sciences, University of California, MercedMerced, CA, USA; ^4^Department of Psychological & Brain Sciences, Dartmouth CollegeHanover, NH, USA

**Keywords:** distribution analysis, bimodality

## Distribution analyses and bimodality

Distribution analyses are becoming increasingly popular in the psychological literature as they promise invaluable information about hidden cognitive processes (e.g., Ratcliff and Rouder, 1998; Ratcliff et al., 1999; Wagenmakers et al., 2005; Miller, 2006; Freeman and Dale, 2013). One particular approach probes distributions for uni- vs. bi-modality, because bimodality often results from the contribution of dual processes underlying the observed data (Larkin, 1979; Freeman and Dale, 2013; see Knapp, 2007, for a historical overview). Although several statistical tools for this purpose exist, it remains unclear which one can be considered as a gold standard for assessing bimodality in practice.

Freeman and Dale (2013) have recently shed some light on the utility of three different measures of bimodality known as the *bimodality coefficient* (*BC*; SAS Institute Inc, 1990), *Hartigan's* dip statistic (*HDS*; Hartigan and Hartigan, 1985), and *Akaike's* information criterion (*AIC*; Akaike, 1974) as applied to one-component and two-component Gaussian mixture distribution models (McLachlan and Peel, 2000). Overall, their analyses favored the HDS but also credited the BC with considerable utility. Notably, however, rather different formulas for the BC can be found in the literature (SAS Institute Inc, 1990, 2012; Knapp, 2007; Bimodal distribution, 2013; Freeman and Dale, 2013)—certainly a potential source of confusion among researchers using the BC.^1^ Additionally, the Appendix of Freeman and Dale (2013) gives a slightly ambiguous formula for the BC because their approach used non-standard MATLAB functions that are not widely accessible. The present article aims at clarifying and correcting these issues in an attempt to prevent misunderstanding and confusion. Further, methodological issues in using this measure are sketched to provide an intuition about its behavior. Note that the current paper does not intend to argue in favor of the BC as compared to other measures (see Freeman and Dale, 2013, for a thorough comparison). Rather, we want to point out pitfalls and limitations of this measure that can easily be overlooked.

## The BC and its caveats

The computation of the BC is easy and straightforward as it only requires three numbers: the sample size *n*, the skewness of the distribution of interest, and its excess kurtosis^2^ (see DeCarlo, 1997, and Joanes and Gill, 1998, for a detailed description of the latter two statistics). First appearing as part of the SAS procedure CLUSTER under the headline “Miscellaneous Formulas” of the SAS User's Guide (SAS Institute Inc, 1990, p. 561), the original formulation of the BC is

$$\text{BC}=\frac{{\text{m}}_{3}^{2}+1}{{\text{m}}_{4}+3\cdot \frac{{\left(n-1\right)}^{2}}{\left(n-2\right)\left(n-3\right)}},$$

with m_3_ referring to the skewness of the distribution and m_4_ referring to its excess kurtosis (see Knapp, 2007, for critical remarks about this notation), with both moments being corrected for sample bias (cf. Joanes and Gill, 1998). The BC of a given empirical distribution is then compared to a benchmark value of BC_crit_ = 5/9 ≈ 0.555 that would be expected for a uniform distribution; higher numbers point toward bimodality whereas lower numbers point toward unimodality.

Freeman and Dale (2013) gave information about computation of the BC with Matlab, but unfortunately two problems likely arise from using their code (for more information and examples of calculation with different software packages, see the online material): First, the call

$${\text{m}}_{3}=\text{skew}(\text{x});$$

likely results in an error, as skew() is not a native Matlab function. The correct call should be

$${\text{m}}_{3}=\text{skewness}(\text{x},\text{0});$$

where the second input parameter 0 prompts the necessary correction for sample bias. Secondly, kurtosis() computes Pearson's original kurtosis (The MathWorks Inc., 2012). To obtain the correct and sample-bias corrected value, the call should be

$${\text{m}}_{4}=\text{kurtosis}(\text{x},\text{0})-\text{3};$$

**Figure 1:**
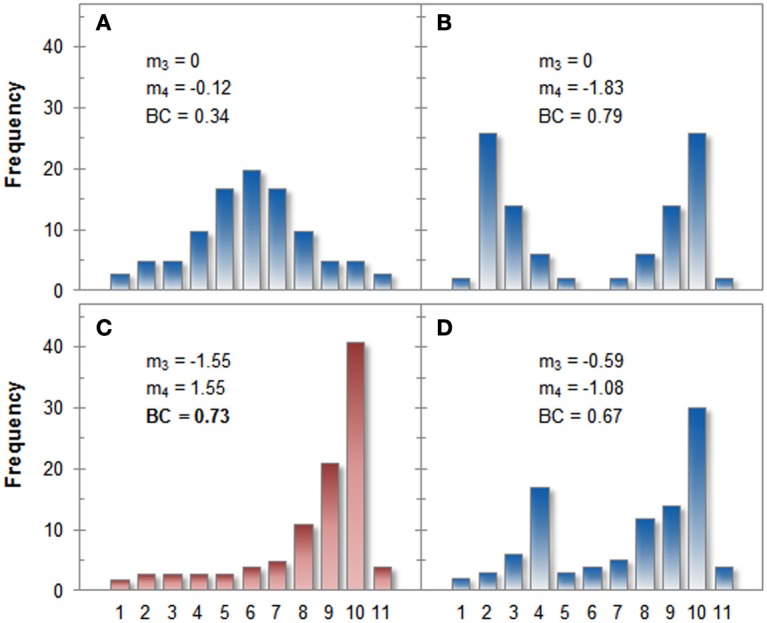
**Histograms for four hypothetical distributions, their skewness (*m*_3_) and kurtosis (*m*_4_), as well as the corresponding BCs (values exceeding 0.555 are taken to indicate bimodality)**. Panel **(A)** shows a clearly unimodal distribution whereas the distribution in Panel **(B)** is clearly bimodal. Both distributions are classified correctly by the BC. Panel **(C)** shows a skewed unimodal distribution that is classified erroneously as bimodal by the BC. The distribution in Panel **(D)** is correctly classified as bimodal, even though its BC is lower than that of distribution C. See the text for a detailed comparison of the distributions.

Irrespective of these computational issues, the above-mentioned formula reveals that the BC is directly influenced by both, skewness and kurtosis: Higher BCs result from high absolute values of skewness and low or negative values of kurtosis. Especially the influence of skewness can result in undesired behavior of the BC. As an illustration, four hypothetical distributions of 100 values each (range 1–11) are plotted in Figure 1, including their skewness, kurtosis, and the resulting BC (see Appendix for the raw data).

Comparing distribution A and B reveals the expected behavior of the BC: The two obvious modes in distribution B decrease kurtosis and increase the BC. Distribution C, however, is clearly unimodal when inspected by eye but its heavy skew also leads to a large BC. In terms of the BC, distribution C is even more bimodal than distribution D even though distribution D clearly has two modes, but otherwise both are very similar. The additional mode, however, decreases skewness thereby lowering the BC as long as it is not compensated by (negative) kurtosis.

## Conclusions

As described above, empirical values of BC > 0.555 are taken to indicate bimodality. A probability density function for the BC, however, cannot be derived (Knapp, 2007). This is a major drawback because it precludes a thorough null-hypothesis significance test.

A suitable alternative test for bimodality is the *dip test* (Hartigan and Hartigan, 1985) that probes for deviations from unimodality (see also Freeman and Dale, 2013, for a more detailed description). An algorithm for this test was proposed after its publication (Hartigan, 1985) and this algorithm has meanwhile been adopted for MATLAB (Mechler, 2002). Additionally, an up-to-date, bug-corrected version was recently published as an R package (diptest, Maechler, 2012).

A direct comparison of the BC and the dip test (Freeman and Dale, 2013) revealed that both measures have merit for assessing bimodality but neither statistic is perfectly sensitive and specific at the same time. Accordingly, one may assess empirical distributions with both measures and diagnose bimodality especially in case of convergent results. Should the results not converge, it seems the best strategy to investigate distributions for other measures, such as skewness and kurtosis individually (as well as their appearance when inspected by eye), to determine whether the result of the BC might be biased in one or the other direction.

## Footnotes

^1^The corresponding Wikipedia article (Bimodal distribution, 2013) used a wrong formula throughout, but has been corrected as part of preparing this article.

^2^Excess kurtosis and Pearson's original kurtosis differ only as to whether the distribution's fourth scaled moment is normalised to a value of 0 for normal distributions or not (with “*excess*” indicating that a value of three has been subtracted for normalisation). The present article assumes all statistics to represent excess kurtosis if not explicitly indicated otherwise.

## Appendix

**Table A1:** **Frequency data of four hypothetical distributions of 100 values each, with corresponding estimates of skewness (m_3_), kurtosis (m_4_), and the BC**.

	**Data Set**			
**Value**	**A**	**B**	**C**	**D**
1	3	2	2	2
2	5	26	3	3
3	5	14	3	6
4	10	6	3	17
5	17	2	3	3
6	20	0	4	4
7	17	2	5	5
8	10	6	11	12
9	5	14	21	14
10	5	26	41	30
11	3	2	4	4
m_3_	0.00	0.00	−1.55	−0.59
m_4_	−0.12	−1.83	1.55	−1.08
BC	0.34	0.79	0.73	0.67

Data set C is adapted from Knapp (2007) (Figure 7).
