# Supplementary material for: Good things peak in pairs: a note on the bimodality coefficient
Source: Front Psychol. 2013 Oct 2;4:700. doi: 10.3389/fpsyg.2013.00700 (PMC3791391; doi:10.3389/fpsyg.2013.00700)
Supplement: Supplementary file 1 [file DataSheet1.ZIP › Supplement/Readme.pdf]

Readme: How to compute the BC for empirical data

As outlined above, the BC originates from the statistical package SAS and, to date, it has not been implemented as a standard method in other dedicated statistical software packages. We thus demonstrate how to compute the BC both, with SAS and when SAS is not readily available. We will give detailed examples for Libre Office Calc, MS Excel, SPSS, Matlab, and R that yield identical results as the SAS procedure `CLUSTER` (see the Supplementary Material for data, demonstration, and syntax files). A major point in computing the BC with these various software packages concerns whether or not bias-corrected estimates for skewness and excess kurtosis can be obtained. Even though Knapp (2007) has explicitly removed bias corrections in his demonstration of the BC, we will focus on the original formulation of the BC (SAS Institute, 1990, 2012) that assumes bias-corrected measures.

Computing the BC with SAS

The easiest way to compute the BC for a given data set is to call the `CLUSTER` procedure of SAS. For this call, we assume the data to be stored in a data set called `bc_data`. This data set includes the four variables A-D which store the raw data relating to the distributions in Figure 1 (100 observations each; see `BC_SAS.sas` in the Supplementary Material for data and syntax). In this case, the BC can be requested with only three lines of code:

```
proc cluster noeigen simple data=bc_data method=centroid;
 var A B C D;
run;
```

The only important keyword in the above code is `simple` which requests a table of summary statistics for each variable. The corresponding output (see the following figure) lists BCs for each variable.

```
                    The CLUSTER Procedure
           Centroid Hierarchical Cluster Analysis

Variable          Mean     Std Dev    Skewness    Kurtosis    Bimodality

A               6.0000      2.2786           0     -0.1174        0.3361
B               6.0000      3.5334           0     -1.8347        0.7947
C               8.3400      2.4339     -1.5472      1.5501        0.7309
D               7.3400      2.8470     -0.5853     -1.0763        0.6657
```

SAS output of summary statistics as requested with a call to the `CLUSTER` procedure (computed with SAS 9.1.3).

Computing the BC with Calc and Excel

Libre Office Calc and MS Excel both offer built-in functions to compute bias-corrected estimates of skewness and kurtosis named `SKEW` and `KURT`, respectively (non-English versions are likely to use different labels). Assuming that the data are entered in the range A1:A100, we can compute the *numerator* of the BC as

```
=SKEW(A1:A100)^2+1 .
```

To compute the *denominator*, we need the sample size which can be computed with the `COUNT` function or, alternatively, simply entered as a numerical value:

```
=KURT(A1:A100)+3*((100-1)^2/((100-2)*(100-3))) .
```

Dividing numerator by denominator gives the BC (see `BC_Calc.ods` and `BC_Excel.xlsx` for demonstration worksheets).

Computing the BC with SPSS

The SPSS function `Descriptives` gives appropriate estimates for skewness and kurtosis if the corresponding boxes are ticked in the options menu. The easiest way to compute the BC, however, seems to be taking these values and computing the BC manually. More sophisticated syntax to compute the BC automatically can be created with the Output Management System (OMS) and a corresponding example is given in `BC_SPSS.sps`.

Computing the BC with Matlab

The appendix of Freeman and Dale (2013) already gave some information of how to implement the BC in Matlab (with the input argument `x` referring to a vector containing the raw data):

```
% BC according to Freeman & Dale (2013)
function [b] = bmtest(x)
     %m3 = skew
     %m4 = kurt
     %n = data size
     m3 = skew(x);
     m4 = kurtosis(x);
     n = length(x);
     b=(m3^2+1) / (m4 + 3 * ( (n-1)^2 / ((n-2)*(n-3)) ));
```

It should be noted, however, that `skew` and `kurtosis` in this code refer to custom-built functions. When using the built-in functions `skewness` and `kurtosis` of Matlab's Statistics Toolbox, one has to be aware of two pitfalls. First, Matlab's native `kurtosis` function does not compute excess kurtosis but rather Pearson's original kurtosis that is not normalised to a value of 0 for normal distributions (The MathWorks Inc., 2012). Accordingly, a value of 3 has to be subtracted from the result of `kurtosis` for all computations. Secondly, `skewness` and `kurtosis` both return estimates that are not bias-corrected by default. This behavior can be controlled by specifying a second input argument that can be set to either 0 (correct for bias) or 1 (do not correct for bias). To match the results of SAS, we recommend using bias-corrected versions. Accordingly, a complete function to compute the BC is described by the following code (see `BC_Matlab.m` in the Supplementary Material for a more detailed example).

```
% Corrected BC
function [b] = bmtest_cor(x)
     % m3 = bias-corrected skewness
     % m4 = bias-corrected kurtosis
     % n = sample size
     m3 = skewness(x,0);
     m4 = kurtosis(x,0)-3;
     n  = length(x);
     b  = (m3^2+1) / (m4 + 3 * ( (n-1)^2 / ((n-2)*(n-3)) ));
```

Computing the BC with R

The current version of R (2.15.2) does not offer built-in functions for skewness and kurtosis but several freely available packages come with the appropriate functionality (e.g., `e1071`, `moments`, `PerformanceAnalytics`, and `psych`). These packages have different algorithms for computing both measures and we recommend using `e1071` (Meyer, Dimitriadou, Hornik, Weingessel, & Leisch, 2012)[1] which allows for specifiying which algorithm is used to compute each statistic and which also gives considerable detail about the implemented algorithms.

Thus, after having installed the package and loading it via `library(e1071)`, we can request unbiased estimates for skewness and kurtosis with the input argument `type = 2` when calling the functions `skewness` and `kurtosis`. When we assume the raw data to be stored in a numeric vector of the name `x`, the BC can be computed in the following four steps (see `BC_R.R` for a more detailed example).

```
m3 = skewness(x,type = 2)
m4 = kurtosis(x,type = 2)
n  = length(x)
bc = (m3^2+1) / (m4 + 3 * ( (n-1)^2 / ((n-2)*(n-3)) ))
```

[1] Meyer, D., Dimitriadou, E., Hornik, K., Weingessel, A., & Leisch, F. (2012). *e1071: Misc Functions of the Department of Statistics (e1071), TU Wien*. R package version 1.6-1. http://CRAN.R-project.org/package=e1071 (retrieved February 11, 2013).
